# Non-straight cell edges are important to invasion and engulfment as demonstrated by cell mechanics model

**DOI:** 10.1007/s10237-015-0697-6

**Published:** 2015-07-07

**Authors:** Matthew C. Perrone, Jim H. Veldhuis, G. Wayne Brodland

**Affiliations:** Department of Civil and Environmental Engineering, University of Waterloo, Waterloo, ON N2L 3G1 Canada

**Keywords:** Cell mechanics, Cell–cell interactions, Computational modelling, Computer simulations, Finite element models, Polyline models, Cell sorting, Cell mixing, Invasion, Checkerboard patterns, Tissue engulfment, Cell engulfment

## Abstract

Computational models of cell–cell mechanical interactions typically simulate sorting and certain other motions well, but as demands on these models continue to grow, discrepancies between the cell shapes, contact angles and behaviours they predict and those that occur in real cells have come under increased scrutiny. To investigate whether these discrepancies are a direct result of the straight cell–cell edges generally assumed in these models, we developed a finite element model that approximates cell boundaries using polylines with an arbitrary number of segments. We then compared the predictions of otherwise identical polyline and monoline (straight-edge) models in a variety of scenarios, including annealing, single- and multi-cell engulfment, sorting, and two forms of mixing—invasion and checkerboard pattern formation. Keeping cell–cell edges straight influences cell motion, cell shape, contact angle, and boundary length, especially in cases where one cell type is pulled between or around cells of a different type, as in engulfment or invasion. These differences arise because monoline cells have restricted deformation modes. Polyline cells do not face these restrictions, and with as few as three segments per edge yielded realistic edge shapes and contact angle errors one-tenth of those produced by monoline models, making them considerably more suitable for situations where angles and shapes matter, such as validation of cellular force–inference techniques. The findings suggest that non-straight cell edges are important both in modelling and in nature.

**Electronic supplementary material:**

The online version of this article (doi:10.1007/s10237-015-0697-6) contains supplementary material, which is available to authorized users.

## Introduction

Demands on computational models are continuing to increase as they play a broader and more central role in the advancement of cell mechanics. These roles now include testing hypotheses about the forces that drive particular motions, pre-testing of proposed experiments, interpretation of experiments, tracing of causal sequences, sensitivity analyses, providing synthetic data for algorithm testing and inspiration of new investigative approaches (Ambrosi et al. 2011; Brodland 2002, 2006; Brodland et al. 2006, 2014; Chen and Brodland 2008; Clausi and Brodland 1993; Davidson et al. 2010; Engler et al. 2009; Grieneisen and Scheres 2009; Hutson et al. 2009; Kabla 2012; Meier-Schellersheim et al. 2009; Ramasubramanian et al. 2006). In some cases, these roles are becoming so significant to the scientific endeavour that it can be difficult to publish experimental results without a complementary computer model.

Over the years, computational models have provided many key insights into the mechanics of cells, and model enhancements have been central to these contributions (Brodland 2004). The cell mechanics models developed during the 1970s (Goel et al. 1970; Gordon et al. 1972) were among the first computer applications, and they provided support for some of the earliest attempts to explain why cells in aggregates underwent sorting, engulfment and a range of other intriguing phenomena (Edelstein 1971; Harris 1976; Steinberg and Wiseman 1972). These models helped to bring order to this branch of science as it struggled to move ahead from its historical and largely phenomenological foundations (Holtfreter 1944; Moscona 1952; Trembley 1744), and they helped to focus attention on the surface properties of cells. Even though the early lattice-based models were rather simple compared to current models, they helped bring structure to the tentative mechanical explanations that were being put forward at the time. In due course, centric and vertex models that allowed cells to take on more-realistic, general polygonal shapes appeared and they brought confidence to the findings of the earlier models, as did sub-cellular lattice-based Potts models (Alber et al. 2003; Glazier and Graner 1993; Honda 1978; Honda et al. 1986).

These models were followed by finite element (FE) models in which the viscosity of the cytoplasm could be included in a mechanically rigorous way (Brodland et al. 2007; Chen and Brodland 2000). These models unexpectedly led to the realization that cell–cell mechanical interactions are driven largely by differences in effective boundary (interfacial) tensions rather than by differences in cell–cell adhesions (Harris 1976; Steinberg 1962). The resulting paradigm shift required some 40 years of experiments, computer simulations and analysis to be re-interpreted.

Although these various kinds of cellular models had visible limitations, they made possible larger-scale models of tissues, organs and whole embryos, and led to fundamental mechanical insights into how genes ultimately drove cell ultrastructure, force generation, tissue mechanics, phenotype and medical outcomes (Ambrosi et al. 2011; Brodland 2006; Brodland et al. 2006, 2010a; Chen and Brodland 2000, 2008; Conte et al. 2008, 2012; Davidson et al. 1999; Engler et al. 2009; Grieneisen and Scheres 2009; Hutson et al. 2009; Kabla 2012; Keller et al. 2008; Lecuit et al. 2011; Meier-Schellersheim et al. 2009; Ramasubramanian et al. 2006; Varner et al. 2010). Over time many of the discrepancies between cell- and tissue-level models and associated experiments were at least partially resolved, often as a result of advances in modelling (Hutson et al. 2008). Unfortunately, model-experiment discrepancies continued to exist, especially with regard to cell shape and contact angles.

**Fig. 1:**
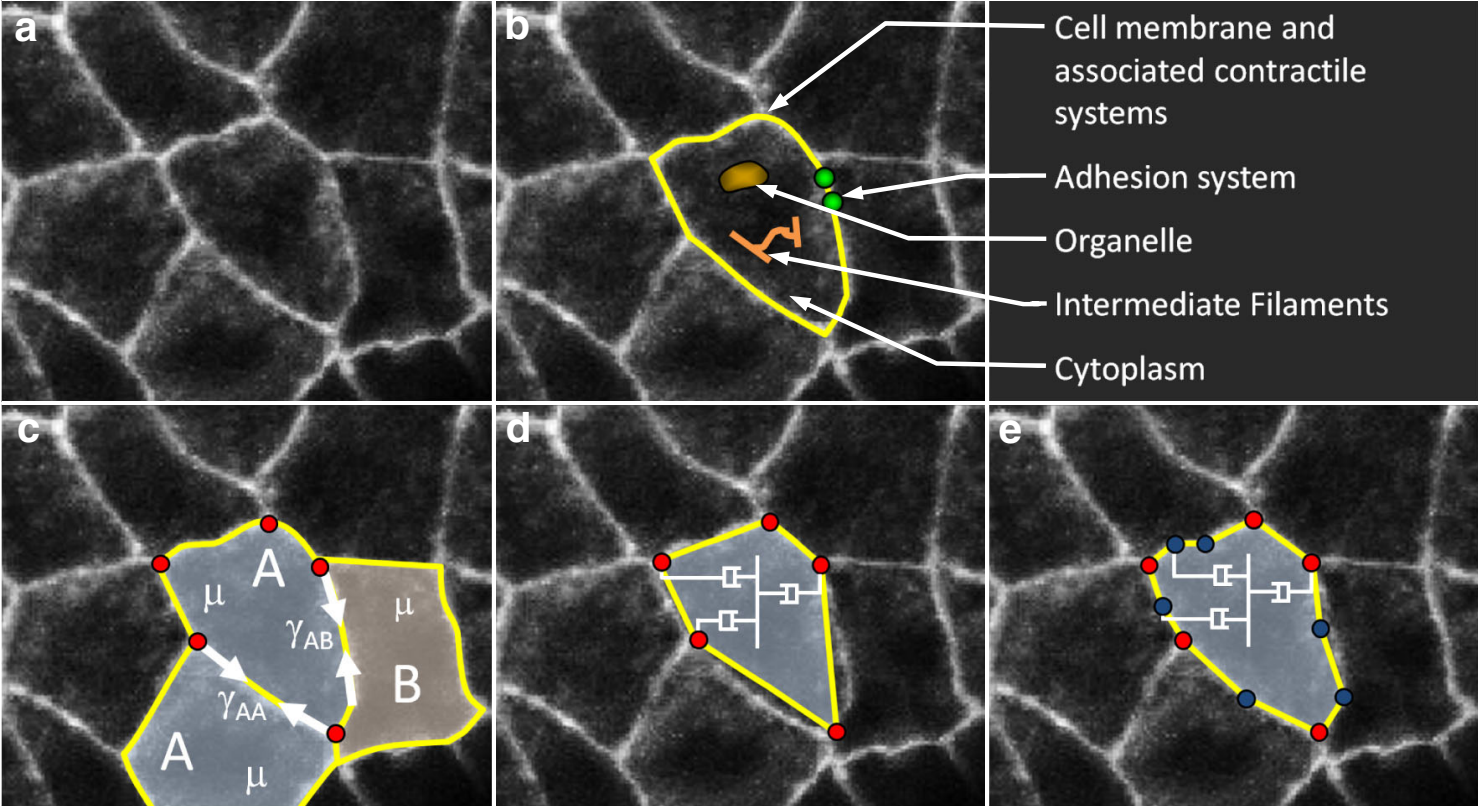
An epithelium and its corresponding models. **a** An image of amnioserosa cells in a *Drosophila* embryo during early dorsal closure (courtesy of M. Shane Hutson). Primary functional and force-generating structures are shown in (**b**). **c** Illustrates the net interfacial tensions $\upgamma$ acting along the cell boundaries and the effective viscosity $\upmu$ of their cytoplasm. For explanatory purposes only, the cells are considered to be of two types and the tensions associated with different kinds of boundaries are labelled with subscripts. **d** Monoline model of the system in (**c**) and straight rod elements (shown in *yellow*) are used to represent each cell edge and to carry its interfacial tension $\upgamma$. Select *dashpots* representing the effective cytoplasm viscosity $\upmu$ are shown. Notice that nodes (*red dots*) exist only at the triple (or higher-order) junctions. **e** The associated polyline model and its segmented edges have multiple rod elements connected by intermediate nodes (shown in *blue*). Note how the segmented edges much more closely approximate the true cell shapes

Virtually all previous cell-level models have approximated the cell–cell edges as being straight, although cell-medium boundaries are often treated as curved or polygonal (Brodland 2004; Graner 1993; Honda 1978; Staple et al. 2010). Modelling internal edges as straight would seem to be a reasonable approach given that cell–cell interfaces are only slightly curved in many situations, including the one illustrated in Fig. 1a.

However, when straight-edge models were pressed into service to provide synthetic (computer-generated) data for testing of nascent force inference techniques, unexpected difficulties arose. The goal of these methods was to infer sub-cellular forces from images. Earlier computational models had shown that cell shape was intimately related to details of the forces that were present, and these models aimed to apply the governing equations in their inverse direction with the idea that forces could be extracted from cell shapes (Brodland et al. 2010b; Cranston et al. 2010). Since the detailed force information needed to test these inverse methods could not be obtained experimentally, synthetic data were generated by running computer simulations in which the driving forces could be arbitrarily specified and the resulting cell shapes known to many digits of accuracy (Brodland et al. 2010b, 2014; Graner 1993; Ishihara et al. 2013). As these force inference techniques were applied to increasingly challenging problems, data from straight-edge models no longer proved satisfactory. This impasse led to the development of a revised force inference approach, one that relied on accurate cell–cell contact angles and interface curvatures, and testing of this new approach required the development of computational models that could generate configurations with edges that were not forced to remain straight. The particular approach we chose represented cell edges as polylines (connected line segments) (Brodland et al. 2014).

The goal of this study is to describe the mathematical and computational foundations of that polyline model; to investigate contact angle, cell shape, and cell motion discrepancies between monoline and polyline models; and to determine whether these discrepancies are a direct result of the straight-edge assumption. We hope, thereby, to address the larger question of whether the mechanical flexibility that curved edges essentially afford to cells facilitates certain kinds of behaviour both in silico and in nature.

To address these objectives, we will compare the results of a polyline model with an otherwise identical monoline (straight-edge) model and develop a detailed mechanical explanation for their differences. We will consider a wide variety of common cell–cell interactions, including annealing, single- and multi-cell engulfment, sorting, and two forms of mixing (invasion and checkerboard pattern formation)—key building blocks for embryogenesis, wound healing, cancer metastasis and tissue engineering. We aim to create a framework that will help modellers choose an appropriate model for studying specific scenarios.

## Formulation of the models

Figure 1a shows a portion of a planar cell aggregate. Its cells derive their mechanical properties from cytoskeletal components and from other structural elements as suggested by Fig. 1b. As argued elsewhere (Brodland 2002; Lecuit and Lenne 2007), the active forces generated by cell membrane and actomyosin contraction, and ameliorated by cell–cell adhesion systems can be combined into a net interfacial tension $\gamma$, which is tangent to the cell boundary (Fig. 1c). In a typical finite element model, this tension is embodied in a single straight constant–force rod element as in Fig. 1d (Brodland et al. 2007; Chen and Brodland 2000). Although straight-line approximations like this are standard in many cell modelling methodologies, they may not represent the cell edge shapes well.

In order to investigate whether inaccuracies introduced by straight-line approximations are important to various cellular phenomena, we also built a model in which cell edges were approximated as polylines (Fig. 1e), that is they were segmented, so that they could more closely approximate the edge shapes of real cells. All rod elements associated with any particular edge were assumed to carry the same tension. The number of intermediate nodes could be adjusted at will, and they were created or removed as the simulation progressed so as to produce segments that were no longer than one-quarter of an average cell diameter. This criterion produced approximately 2 or 3 intermediate nodes per edge on average and was found to produce motions and shapes essentially the same as those that had more, suggesting that shape convergence had been achieved. Although this length criterion worked well for partitioning the polyline segments, one might also base it on local curvature or some other criterion. Edge length works well because longer edges, which will naturally have greater angular changes from one of their ends to the other, will contain more polyline segments. As in previous finite element models, nodes that might otherwise connect four or more cell edges together—quad junctions and rosettes, respectively—were approximated by closely spaced triple junctions. The monoline model can be considered a special case of the polyline model, wherein the maximum edge segment length is set large enough that no intermediate nodes are introduced. For this reason, and because the monoline model has been described elsewhere (Chen and Brodland 2000; Brodland et al. 2007), its detail is not discussed further here.

The cytoplasm, organelles and filamentous networks inside each cell are assumed to play a passive role and to generate an effective viscosity $\upmu$ (Fig. 1c), which is modelled using an orthogonal dashpot system, part of one set of which is shown schematically in Fig. 1d, e (Brodland et al. 2007). The procedure for calculating the dashpot coefficients from the cell geometry and viscosity $\upmu$ is reported elsewhere (Brodland et al. 2007). When intermediate nodes are used, the denominator used in that calculation must contain not the number of nodes n in the cell as in Eqs. 8 and 9 of that study, but the number of triple junction nodes plus half of the total number of intermediate nodes. That the intermediate nodes should be weighted by one-half was determined using patch tests (Irons and Shrive 1983), and this result was found to be appropriate for cells with up to 10 intermediate nodes per side on average, cytoplasm that was not necessarily incompressible (i.e., not restrained to a Poisson’s ratio of $\upnu =0.5$), and aspect ratios (Brodland et al. 2006) as high as 4 (well beyond the normal range of cell shapes). A definitive analytical argument for this experimentally determined weighting factor was not identified.

Both models were run for a fixed number of time steps of specified size. For each time step, the vector sum of the interfacial tensions (rod tensions) framing into each node is calculated and assembled into a global force vector ***f*** as reported elsewhere (Chen and Brodland 2000). The effects of the dashpots in each cell are likewise assembled to produce a global viscous matrix ***C***. The incremental displacement of each node ${\varvec{\Delta }}{\mathbf {u}}$ during a time step $\Delta t$ is then calculated by solving the matrix equation

1 $$\begin{aligned} {\mathbf {f}}=\frac{1}{\Delta t}{\mathbf {C}}\,\Delta {\mathbf {u}}. \end{aligned}$$

An updated Lagrangian approach is used, and so the locations of the nodes, and the ***f*** and ***C*** matrices are updated with each time step (Chen and Brodland 2000). In carrying out these calculations, the intermediate nodes in the polyline model are treated in exactly the same way as the triple junction nodes. Volume constraints on each cell are implemented through Lagrange side conditions (Chen and Brodland 2000).

As the model runs, some cell edges may become quite short, and, consistent with real cells, we assume that they change neighbours (Chen and Brodland 2000; Eaton and Julicher 2011; Walck-Shannon and Hardin 2014). When a boundary between two cells in the model reaches a specified minimum length $\updelta$, typically 2.5 % of an average cell diameter, that boundary will be eliminated and a new boundary will be created between the two cells that were previously separated by the short edge length. The new boundary is given a length longer than the specified minimum to ensure that that neighbour change does not reverse in the next time step (Chen and Brodland 2000). An embargo timer is also sometimes used, as in the tissue engulfment studies reported here, to prevent any new edges with high tensions from spontaneously shortening and changing back.

The length of individual polyline segments also changed as the model ran, and it was often necessary to adjust their number from one time step to the next. When the length of any particular polyline segment was larger than the user-specified maximum, typically 25 % of a cell diameter, it was divided into two line segments of equal length with a new intermediate node between them. Should a particular polyline segment become smaller than a specified minimum length, typically 2.5 % of a cell diameter, the two nodes at its ends were merged into one node. If both nodes were intermediate nodes, the new node was placed in the middle of the old line segment. If one was an intermediate node and the other a triple junction, the intermediate node was simply merged with the triple junction node. If both nodes were triple junctions, that edge is assumed to be governed by the neighbour change criterion described above. All models were verified using patch and convergence tests (Irons and Shrive 1983).

**Fig. 2:**
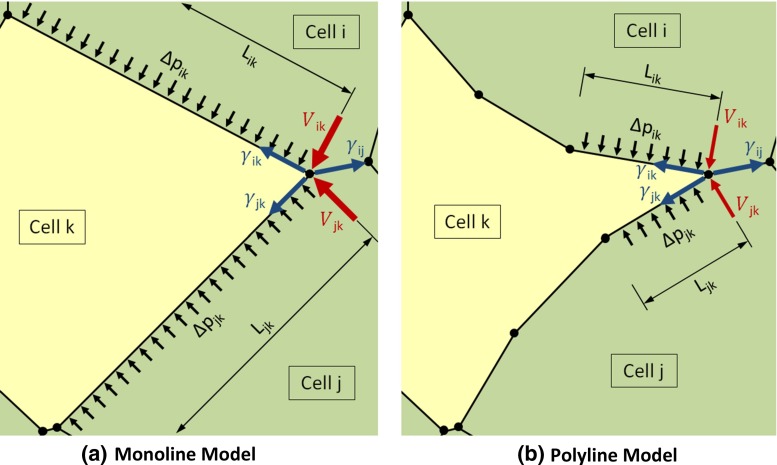
How monoline edges restrict motion. The edge tensions in the figure were chosen so that they should pull the yellow cell between the two green cells (Brodland 2002), though the lengths of the tension vectors do not reflect their relative magnitudes. However, when cell edges are forced to remain straight, as in (**a**), intracellular pressure differences $\Delta \hbox {p}_\mathrm{ab}$ can act over a long enough length $\hbox {L}_\mathrm{ab}$ that the equivalent shear forces $\hbox {V}_\mathrm{ab}$ they generate (Eq. 2) and transmit to the triple junction (in this case between cells *i*, *j* and *k*) can prematurely arrest its motion. When a polyline model is used (**b**), the segment lengths $\hbox {L}_\mathrm{ab}$ are shorter and the resulting shear forces have a much reduced effect

As Fig. 2 suggests, the basic mechanics of the two models is actually quite different (Brodland et al. 2014). When cell edges are forced to remain straight, they behave essentially like beams and transfer the intracellular pressure loads to their ends, where the transverse shear

2 $$\begin{aligned} \hbox {V}_\mathrm{ab} =\frac{1}{2}{\Delta } \hbox {p}_\mathrm{ab} \hbox {L}_\mathrm{ab} \end{aligned}$$

at the beam ends, which is proportional to the intracellular pressure difference $\Delta \hbox {p}_\mathrm{ab}$ and the side length $\hbox {L}_\mathrm{ab}$, must be added to the vector sum of the beam tensions ${\upgamma }_\mathrm{ab}$ to obtain the total triple junction loads (Fig. 2a). When an edge is made of multiple segments (Fig. 2b), the shear loads become a fraction of the value found in monoline models since the lengths $\hbox {L}_\mathrm{ab}$ decrease. As the number of segments is made larger, the shear forces $\hbox {V}_\mathrm{ab}$ decrease until, in the limit as the segment lengths approach zero length, they disappear. The edge tensions in Fig. 2 were chosen to make the yellow cell draw in between the two green cells, and with a polyline model they do. However, in the monoline model, the elevated shear loads $\hbox {V}_\mathrm{ab}$ associated with its substantially longer edges $\hbox {L}_\mathrm{ab}$ are sufficient to counteract the triple junction tension imbalances, blocking further rightward motion of the triple junction between cells *i*, *j* and *k*, and obstructing invasion of the yellow cell. Another effect of a polyline model is that the intermediate nodes displace laterally with respect to the boundary. They move until the tensions along adjacent segments form an angle at which the transverse components of the edge tensions just balance the pressure forces, like a discretized membrane.

## Studies of cell–cell interactions

### Annealing

The first scenario we consider here is annealing of an isolated group of cells of a single type, but with intentional size variations so that pressure differences exist between neighbouring cells (Table 1, Row A). The initial configuration is a Voronoi tessellation (Chen and Brodland 2000), an arrangement commonly used as the starting point for cell models, including all of the models presented here. All edges were assumed to produce the same edge tension $\upgamma$ and, in accordance with equations like those for a pressure vessel (Boal 2012; Viens and Brodland 2007), the pressures that result in the smaller cells are higher than those in the larger cells. This pressure differential produces bulging of the cell edges, with the resulting curvature in any particular edge being proportional to the pressure difference across it, as described by the Young–Laplace equation (Brodland et al. 2014). Spacing the intermediate nodes according to polyline segment length rather than having a fixed number of intermediate nodes per cell edge allows the longer and substantially curved exterior surfaces to have more intermediate nodes than the shorter interior edges.

**Table 1:** A summary of the simulations

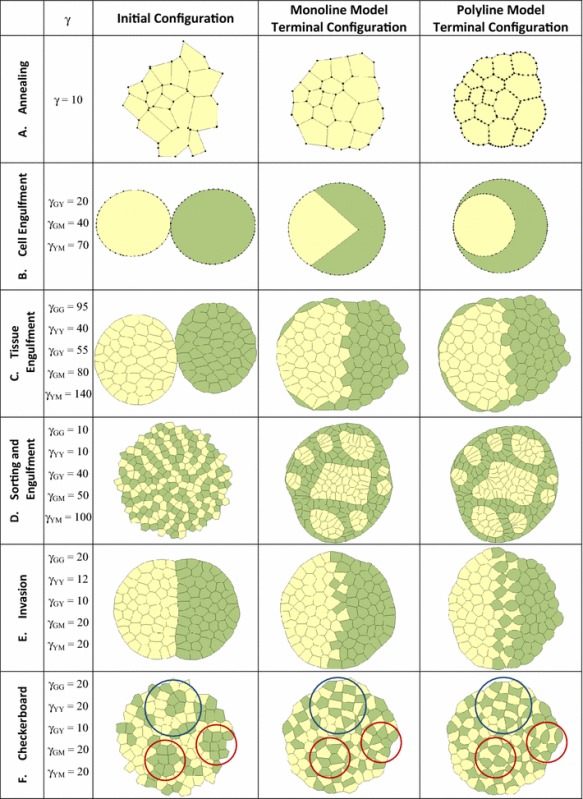				

The title of each scenario is shown in the first column of the table, and the surface and interfacial tensions used for each are reported in the second column. The middle column gives the initial configuration for both models, and the terminal states (the geometries at which further meaningful movement ceases) for the monoline and polyline models are shown in the second last and last columns, respectively

As the monoline and polyline models ran, they produced topologies that were similar, but not always the same. The terminal configurations of the monoline and polyline models are shown in the last two columns of Table 1, and they represent equilibrium configurations in which all meaningful motion has stopped (see also Movie 1). Unlike the monoline model, the polyline model is able to produce cell edge shapes consistent with those seen in Fig. 1a and many other published studies (Eisenhoffer et al. 2012; Maitre et al. 2012; Solon et al. 2009). Polyline models like the one in the first row of Table 1 played an important role in development and validation of the CellFIT force inference technique (Brodland et al. 2014). As one can see, the polyline model gives contact angles that are different from those that the imposed straight edges of the monoline model produces. In order to quantitatively assess the angles produced by these two kinds of models, RMS errors between them and corresponding theoretical Young angles (Davies and Rideal 1963) were calculated for each triple junction. For the monoline model, these errors were distributed essentially uniformly from 2.5$^{\circ }$ to 40$^{\circ }$, with a mean of 19.5 and a median of 20.7$^{\circ }$. Although these errors may not significantly affect the appearance of the angles, they are enough to complicate edge force calculations (Viens 2006) and significantly disrupt inverse methods that strive to infer edge tensions from shape.

**Fig. 3:**
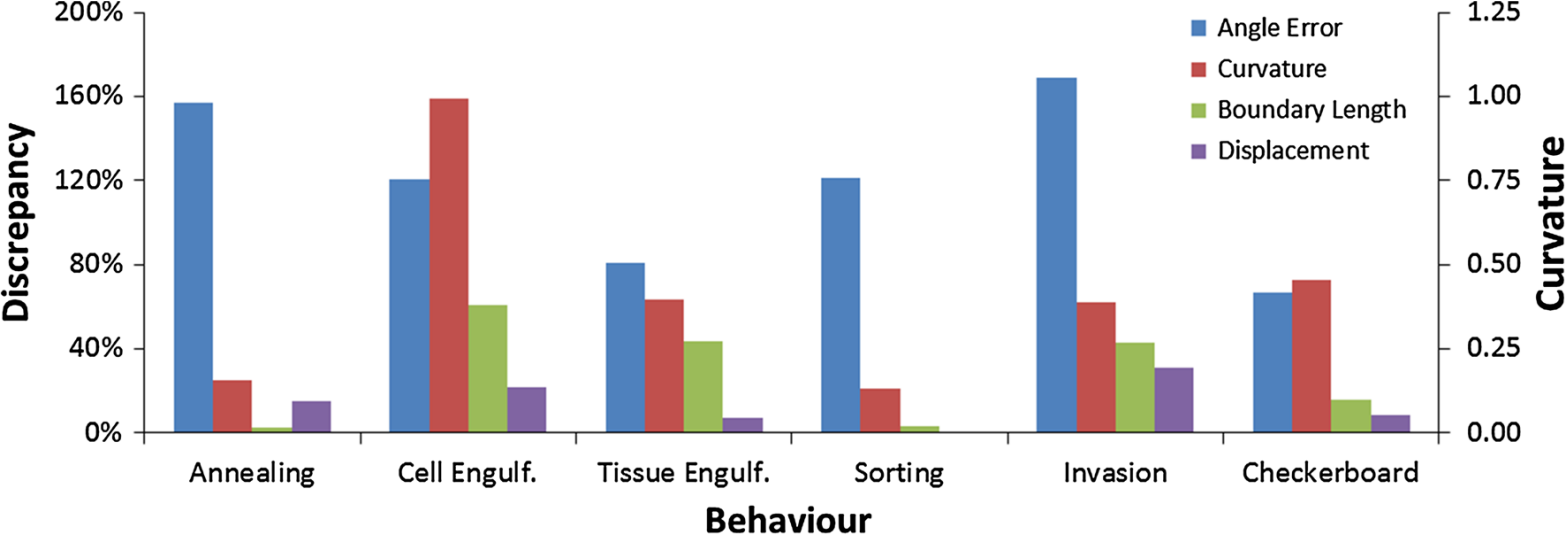
A comparison of monoline and polyline models. The models are compared in terms of angle error, curvature, boundary length and displacement for each cell interaction scenario. Details of the calculations are given in the text and may vary from one scenario to the next, depending on the particular features of interest. All values are reported as percentage differences between the monoline and polyline models (*left ordinate*), except for curvature (*right ordinate*)

In contrast, when circular arcs are fit to the multiple points along each edge of a polyline model (Brodland et al. 2014), the RMS angle errors range from 0.04$^{\circ }$ to 2.8$^{\circ }$ with one outlier at 9.0$^{\circ }$. Most triple junction errors were under 1$^{\circ }$, the average RMS error was 1.2$^{\circ }$ and the median value was 0.69$^{\circ }$. These errors represent a dramatic improvement over those of the monoline model, and they are well within the range needed for successful application of force inference methods. Figure 3 shows a single discrepancy percentage, which is an integral over time, except for curvature, which because the monoline model gives only zero values, is calculated at the terminal configuration. The reported discrepancy percentages are equal to the difference in the monoline and polyline values divided by their mean.

As shown in Fig. 3, the mono and polyline models can also be compared in terms of cell shape, as described by average curvature of the internal cell edges. In annealing simulations, the curvature is small and little difference is seen between the mono and polyline models there. The total edge length of a specific kind, in this case the internal edges, can also be a useful indicator of the state of an aggregate, as can the average distance between the initial and final locations of the cell centroids. Although the latter measures do not reveal meaningful model differences in annealing scenarios, they are informative in some of the others we consider.

### Cell engulfment

Next, we consider a group of simulations that involve two cell types (Brodland 2004; Brodland and Chen 2000; Brodland 2002), henceforth identified by their assigned yellow and green colours. The simplest such simulation involves one cell of each type, and here the tensions have been chosen so that one cell should totally engulf the other (Brodland 2002). This simulation generates complex cell shapes and reveals significant differences between the monoline and polyline models.

Two examples where cell engulfment occurs in nature include complete engulfment of apoptotic cells by phagocytes (Ravichandran and Lorenz 2007) and partial or complete engulfment of beads and other textured coatings applied to orthopaedic and dental implants by cells, a process wherein the curvature of the engulfed particles can be a limiting factor (Tache et al. 2004).

For engulfment as shown in Row B of Table 1 and Movie 2 to occur, sufficient tension must exist along the yellow-medium boundary that it can pull the green cell over the surface of the yellow cell. For this to happen, the sum of the tensions along the green-medium interface and the heterotypic interface must be less than the yellow-medium tension (Brodland 2002). As the figure in the monoline model column demonstrates, straight intercellular edges severely restrain these motions and, at best, partial engulfment and unrealistic cell shapes can be obtained. Engulfment is ultimately blocked by the virtual shear forces described in connection with Fig. 2a, and these forces cause the Young–Laplace equation to be violated at the triple junctions along the aggregate perimeter.

In contrast, when the intracellular boundary is a polyline, it can wrap around the yellow cell, forming a crescent shape, and ultimately engulfing it (last image in Row B of Table 1). This example shows that the added compliance afforded by polyline edges gives them a major advantage over monoline edges and it demonstrates a phenomenon in which the ability of the cell surface to curve is fundamental. The significant differences between the models are reflected in the increased heights of the associated discrepancy measures in Fig. 3, which reports the curvature of the internal cell–cell boundary, the length of the yellow-medium boundary and the centroidal displacements.

A general principle in modelling is that the shape functions, basis functions, or other factors that control the deformations in the model should allow them to “conform” as much as possible to the natural motions of the system (Zienkiewicz and Taylor 2005). That is, the model should not be prevented from moving like the natural system because of imposed constraints (such as enforced straight edges). If two models are available and the more complex one is able to undergo all of the motions of the simpler one—as is the case in the polyline model as compared to the monoline one—then the more complex model can be trusted to be closer to the truth. It can also serve to identify the range within which simplified models are appropriate to use (Brodland 1988).

### Tissue engulfment

Closely related to engulfment of one cell by another is engulfment of one tissue by another, and it has also been studied extensively (Armstrong 1989; Foty et al. 1996; Phillips and Davis 1978; Steinberg 1963; Steinberg and Wiseman 1972; Steinberg 1962, 1970). One might think that this process would be easier to orchestrate, but it turns out that it is not, because as seen in Row C of Table 1 and Movie 3, the tensions present along the green–green interfaces must be high enough that individual cells can be drawn away from the green mass and flow over the outside of the yellow cells (that is, high enough that the longer, initial boundaries between them and the green mass can shorten), but not so high that the green cells pinch off from each other or pull the yellow cells between the green cells. Tissue engulfment is one of the scenarios where the additional neighbour change embargo timer was needed because without it the high interfacial tension of a newly formed boundary can be much larger than that of the surrounding interfacial tensions, resulting in the new boundary shortening quickly and triggering a new neighbour change that undoes the one that just happened.

The ability of polyline cells to form shapes that are more sinuous allows them to better engulf cells of another type. The heterotypic interfacial boundary looks rather similar for both models, which is not surprising since the cell shapes along this boundary are not complex. However, the polyline model demonstrates complete engulfment while the monoline model produces only partial engulfment. The difference between the two models arises because the engulfing cells in the polyline model can accommodate more complex shapes, stretch further around the engulfed tissue and better separate from the original mass of homotypic cells. Three green cells have dissociated from the bottom edge of the green mass in the polyline model and formed a string of engulfing cells, while only two dissociated in that region in the monoline model. The next cell that would have dissociated was restrained from doing so by its shape constraints. As this simulation demonstrates, a polyline model is more appropriate for tissue engulfment studies. Because the heterotypic boundaries associated with tissue engulfment, as opposed to cell engulfment, involve multiple cells, they can be non-straight even though the edges of the cells from which they are formed may be straight. As a result, the discrepancies between monoline and polyline models are less in this scenario than in the former one (Fig. 3). The discrepancies are calculated on the same basis as the cell engulfment case, except that the displacements are calculated only for cells on the heterotypic interface.

### Sorting and engulfment

One of the intriguing characteristics of embryonic cells in heterotypic aggregates is that they can spontaneously sort out from each other by type (Foty et al. 1996; Graner 1993; Harris 1976; Hutson et al. 2008; Krens and Heisenberg 2011; Maitre et al. 2012; Moscona 1952, 1957; Steinberg 1963; Townes and Holtfreter 1955). Computer simulations showed that the ordering of the final configuration, that is which cells are engulfed and left to form islands or “inclusions” inside, and which cells form the “matrix” phase and cover the outside of the mass, is governed by the surface tensions of the cells (Brodland and Chen 2000; Brodland 2002; Green 2008). Thus, when considering finite aggregates, that is ones with edges that contact medium, sorting and engulfment must be considered together (Armstrong 1989).

Row D of Table 1 and Movie 4 show an initial configuration consisting of 200 cells of two types. The tensions acting along each interface type were chosen to produce cell sorting and engulfment (Brodland 2002). Both models show similar terminal states (last two columns of Row D) in terms of the number, sizes and shapes of the homotypic islands produced, and the motions of both are consistent with those observed in the experiments noted above. The high degree of similarity between the models, except for angle error (Fig. 3), is possible because the shapes of the cells during the sorting process are compact and simple, and both models can adequately replicate these shapes. The engulfment aspects of the process that take place along the aggregate surface produce more complex cell shapes, and transient differences arise between the two models.

**Fig. 4:**
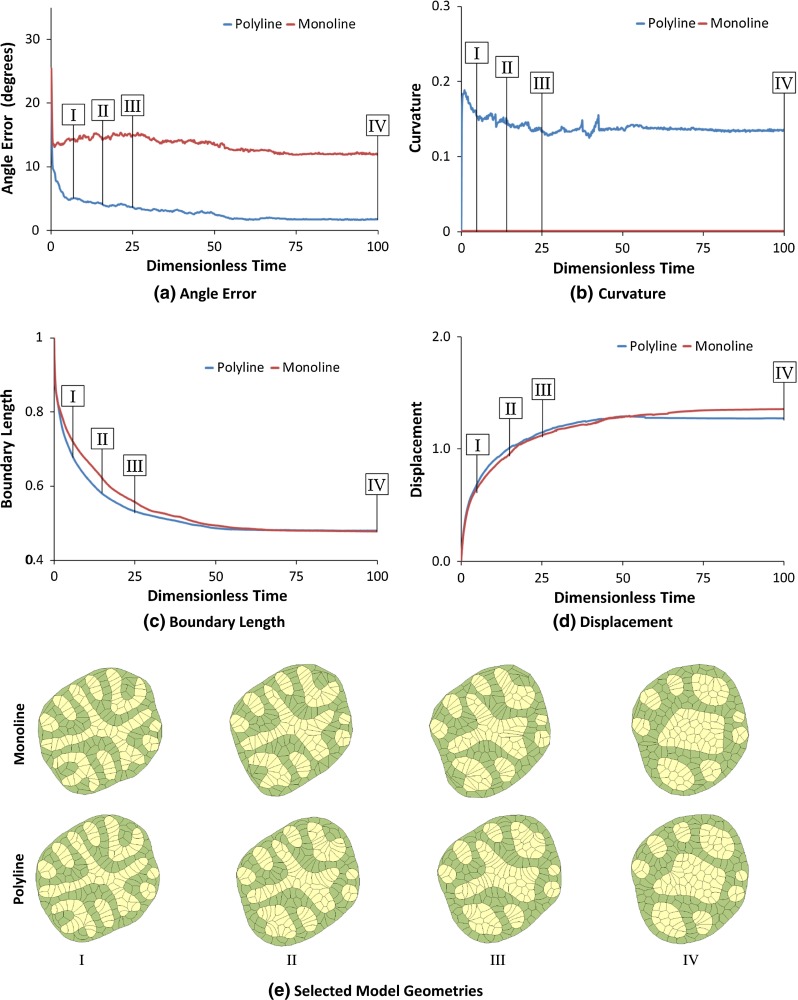
A closer look at cell sorting and engulfment. **a** How median RMS angle error (see text) changes with dimensionless time (Brodland et al. 2006). **b** The average absolute value of edge curvature normalized to a circle of the same area as the average cell. **c** The total length of the yellow–green boundary normalized to its initial length. **d** Displacement (see text for details) normalized to cell diameter. **e** The geometries of the aggregates corresponding to the Roman numerals on the graphs

As Fig. 4a shows, the median angle error in the poly line model is one-third or less of that associated with the monoline model and reduces to one-seventh of the monoline value when dimensionless time reaches 100. The mean curvature along the heterotypic cell–cell interfaces (Fig. 4b) is modest and relatively steady. Both models give similar total heterotypic interface length (Fig. 4c), but that length decreases somewhat more quickly in the polyline model, suggesting that its cells are somewhat more compliant. Finally, Fig. 4d shows that the cells along the heterotypic boundary move to similar degrees.

### Invasion

Whereas sorting causes cells to preferentially contact cells of their own kind, a variety of mixing-like scenarios involve the opposite. In mixing, these forces cause cells from one homotypic group to partially or completely leave that group and mingle with cells of another type. When single cells leave a group, the process is called invasion. In all of these cases, heterotypic interfaces are energetically preferred over the homotypic ones (Brodland 2002), and the interfacial tensions shown in the second column of Table 1 reflect these differences (Brodland 2002). From a mechanical point of view, invasion is a complex phenomenon, involving interactions between the invading cell and at least two neighbouring kinds of cells.

These mixing-related behaviours are important to a number of biological processes. For example, the interface between mesenchymal and myocardial tissues of a developing avian heart is dispersed, that is the two tissues forming the border intermingle as a result of mixing along their common border (Armstrong and Armstrong 1990). Epithelial cells demonstrate invasive behaviour during wound healing (Brugues et al. 2014), and endothelial cells exhibit similar behaviour during angiogenesis (Armstrong and Armstrong 2000). In addition, invasion is the signature behaviour of malignant tumours and the primary step in cancer metastasis. Cancer cells that have previously resided within a well-confined primary tumour invade the surrounding stroma, a first step towards the eventual colonization of a secondary tumour (Valastyan and Weinberg 2011).

Row E of Table 1 and Movie 5 show invasion, and the interfacial tensions in these simulations were set such that the yellow cells invade the green cells. The interfaces between the green cells have relatively high tensions so that they shorten when possible and the adjacent yellow cells have relatively low surface tensions so that they can be drawn in between the green cells. As the figures show, the yellow cells of the polyline model were pulled substantially further into the green cells than were their monoline counterparts. Once again, restrained motion is observed in the monoline model, evidently because of induced equivalent shear forces as in Fig. 2. The more complex shapes of the invading yellow polyline model cells indicate that they deform in a mode different from what the monoline cells allow. Consequently, polyline models must be used to model invasion because of the complex shapes that the invading cells acquire. It should be noted that the yellow cells stop advancing once the trailing parts of them are covered completely by green cells that they have migrated past. At that point, they are trying equally hard to advance into the cells in front of them and behind them, and so their motion is arrested. For cells to move more than one cell diameter, or two if cell positional exchanges occur, as in this case, other mechanisms must come into play.

**Fig. 5:**
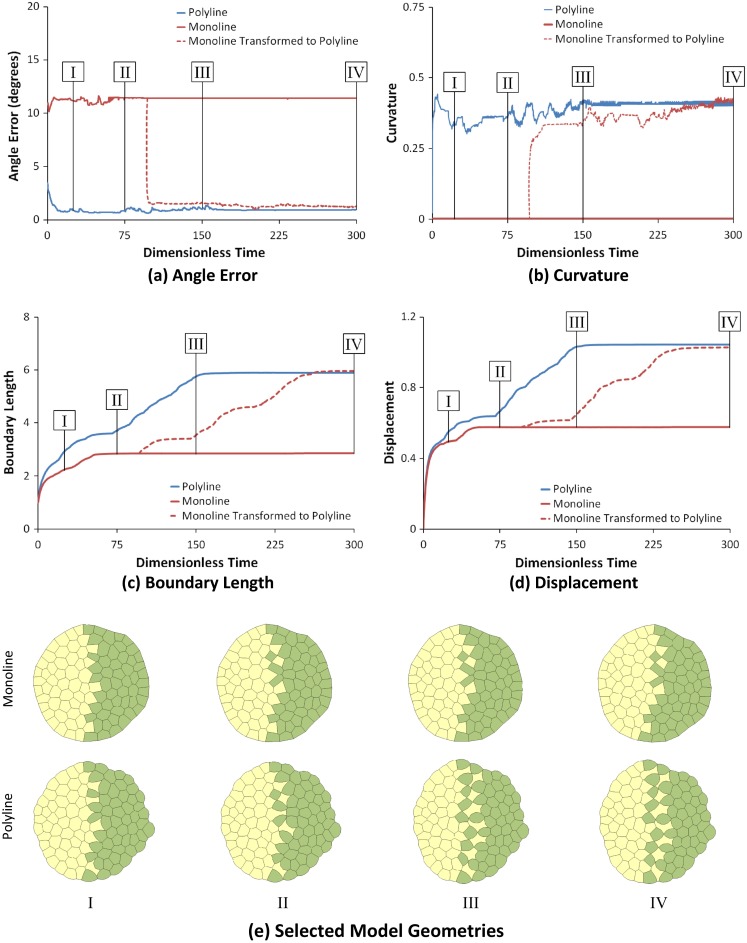
A closer look at invasion. As in Fig. 4, **a** shows median RMS angle error, **b** gives edge curvature, **c** reports the normalized total length of the yellow–green boundary and **d** shows the normalized horizontal displacement. **e** The geometries corresponding to the Roman numerals on the graphs

As Fig. 3 shows, the polyline invasion model is superior to the monoline model in a number of important ways, and Fig. 5 provides additional information about why this is the case. Part (a) shows that the polyline angle errors are dramatically smaller than those of the monoline model. Furthermore, when the monoline geometry at dimensionless time 100 is converted into a polyline model (dashed red curve), its angle error drops rapidly. As the yellow cells invade further into the green mass, the number of quad junctions that are approximated as two closely spaced triple junctions, increases, and this limitation of the code interferes somewhat with the contact angles, but does not meaningfully affect the median angle reported in the figure. When the monoline model is transformed into a polyline model, the curvature of its yellow–green edges (Fig. 5b), yellow–green boundary length (Fig. 5c) and the displacements of cells that began at that interface (Fig. 5d) also rapidly approach those of the polyline model. The cell shapes revealed by the polyline model provide a wealth of important information that is not contained in the monoline model. For example, the polyline model reveals easily identified shape differences between the yellow and green cells. The model shows that when the green–green interfaces have elevated tensions, the yellow cells are drawn in and acquire distinctive star-like shapes, the kind of paradoxical findings that computer simulations are ideal for identifying. Thus, the cell shapes indicate which cell type had the atypical properties (the yellow ones, in this case).

These findings suggest that polyline models may have an important role to play in the quest to understand invasion of cancer cells into surrounding stroma and in potential clinical assays to assess the mechanical reasons that they are able to advance in particular settings. This idea is also consistent with those that underlie CellFIT (Brodland et al. 2014).

### Checkerboard

The formation of checkerboard patterns can be considered an extreme example of cell mixing, where heterotypic interfaces are strongly favoured over both homotypic ones. Though less common than invasion, it does occur naturally on the luminal surface of the oviduct epithelium of the adult Japanese quail. There, the epithelium is a monolayer cell sheet consisting of two types of columnar cells: ciliated cells and gland cells. During sexual maturation, they assemble into a checkerboard-like pattern from a star-like one (Honda et al. 1986).

Row F of Table 1 and Movie 6 show the formation of checkerboard patterns. Both models show generally similar results in terms of the location of the cells and their overall degree of checkerboard patterning. However, differences appear when examining how well the homotypic regions separate. The blue circle outlines an area where both models produced similar results, while the red circles indicate areas where the polyline model outperforms its monoline counterpart. The reason for the discrepancies is that the cells must take on complex shapes in order to separate in these regions and the monoline model is restrained because it lacks the required shape compliance. Thus, although the monoline model can sometimes produce checkerboard motions, the polyline model is more robust in terms of shape compliance. As Table 1 and Fig. 3 show, the monoline model fails to provide good angle estimates, and reproduce the significant cell edge curvatures that arise along the heterotypic boundaries, although it adequately reproduces the heterotypic boundary length and centroidal displacements.

## Discussion and conclusions

Ever since the first computational models of cell mechanics were constructed in the 1970s, straight edges have been considered a suitable approximation to the actual shapes of cell edges, appearing in many kinds of models, from cell lattice models to centric models to vertex models to finite element models (Brodland 2004). As the present study shows, cells with straight edges are subject to artificial shape constraints that can limit both their deformations and motions. In contrast, the cell shapes produced by polyline models can be fluid and as realistic as those produced by some of the best centric models (Brodland 2004), and they have the added advantage that their shapes are based entirely on edge force mechanics. Thus, they bring together the mechanical strength of finite element models, the curved-edge advantages of centric models and the range of cell shapes available in Potts models.

One of the characteristic features of cellular media is the discrete nature of the neighbour changes that occur. These contact exchanges affect the mechanics (Blanchard et al. 2009; Brodland et al. 2006; Chen and Brodland 2000; Eaton and Julicher 2011), and they cause topological differences (bifurcations) to arise between models that are otherwise insignificantly different from each other. Differences of this type can be seen in the sorting simulations (Row D of Table 1), where cells in the upper right island of the monoline model have been pinned more strongly to the yellow–green interface than the corresponding cells in the polyline model, due to lack of neighbour changes along that edge. Along the upper edge of the large central island, the polyline model shows extra pinning. As this example shows, care must be exercised in interpreting the models, and the full history and detailed mechanics must be taken into account.

As part of our convergence checks, models with increased numbers of intermediate nodes were run, and when cell edges contained approximately, three segments on average, the cells underwent all phenomena (except single cell engulfment, which is a special case), nearly as easily and completely as models with more segments per edge. Increasing the number of edges increases computer run times. For example, a model with 10 intermediate nodes per edge on average takes approximately 100 times longer to run than the comparable monoline model. Thus, the discretization used here provided a favourable solution in terms of precision and run times.

As the angle studies showed, a polyline model with circular arc curve fitting typically provides significantly better angle approximations than does a monoline model. Triple junction angles can also be calculated using the polyline segment closest to the junction (Brodland et al. 2014), and polyline models with 2, 3, 4 and 10 segments per edge on average produced corresponding errors of 11.5$^{\circ }$, 8.2$^{\circ }$, 6.1$^{\circ }$ and 3.0$^{\circ }$. The latter error is quite acceptable as synthetic data for verification of current force inference techniques, but curve fitting to edges with four segments per edge provides even lower errors, and faster run times. Scenarios such as checkerboarding and invasion can produce relatively high numbers of quad and higher-order junctions, and model enhancements that would allow them to be handled directly rather than being replaced with compound triple junctions would improve angle reliability and further reduce overall angular error.

This study suggests that monoline models—whether finite element, vertex or centric in formulation—may be suitable for modelling movements where cells remain largely isotropic in shape and where cells are not pulled between or around other cells. Examples would include annealing, cell sorting or checkerboard patterning. Perhaps one of the reasons that the need for polyline models has not been identified earlier is that many computational models have focussed on exactly these behaviours. However, even in these scenarios, monoline models have much higher angle errors than do polyline models with curve fit edges. Only the latter provide data that are suitable for verifying force inference techniques like CellFIT (Brodland et al. 2014). Polyline models are also necessary when complex cell shapes occur—as during invasion and engulfment—two behaviours prominent in breast cancer invasion and other problems of current interest. The study also raises a more philosophical observation, namely that the membrane that envelops animal cells must be flexible in bending in order to facilitate a wide range of reshaping and rearrangement behaviours that are crucial to embryogenesis, a host of normal and disease processes, wound healing and tissue engineering.

This study has described the mathematical and computational foundations of the polyline model; investigated cell contact angles, edge curvatures, edge lengths and cell motions; shown that a variety of known cell behaviours are significantly impeded when cell–cell edges are forced to remain straight; and explained the mechanical reason that this is the case (Fig. 2). Our hope is that this study will provide a framework that can assist modellers to select an appropriate model.

## Electronic supplementary material

Movie 1. Cell Annealing, as in Table 1 Row A (AVI 382 kb)

Movie 2. Cell Engulfment, as in Table 1 Row B (AVI 574 kb)

Movie 3. Tissue Engulfment, as in Table 1 Row C (AVI 4052 kb)

Movie 4. Sorting and Engulfment, as in Table 1 Row D (AVI 8200 kb)

Movie 5. Invasion, as in Table 1 Row E (AVI 3178 kb)

Movie 6. Checkerboard, as in Table 1 Row F (AVI 1960 kb)
